# Methylsulfonylmethane sensitizes endometrial cancer cells to doxorubicin

**DOI:** 10.1007/s10565-020-09542-4

**Published:** 2020-06-20

**Authors:** Karolina Kowalska, Dominika Ewa Habrowska-Górczyńska, Dominika Kurczewska, Kamila Domińska, Kinga Anna Urbanek, Agnieszka Wanda Piastowska-Ciesielska

**Affiliations:** 1 Medical University of Lodz, Department of Cell Cultures and Genomic Analysis, Zeligowskiego 7/9, 90-752 Lodz, Poland; 2 Medical University of Lodz, Faculty of Medicine, Lodz, Poland; 3 Medical University of Lodz, Department of Comparative Endocrinology, Lodz, Poland

**Keywords:** Methylsulfonylmethane, Endometrial cancer, Doxorubicin, Apoptosis

## Abstract

**Background:**

Methylsulfonylmethane (MSM) is a commonly used diet supplement believed to decrease the inflammation in joints and fastens recovery in osteoarthritis, gastric mucosal injury, or obesity-related disorders. It was also suggested that MSM might play a beneficial role in cancer treatment.

**Purpose:**

So far, the MSM might have a potentially beneficial effect in endometrial cancer (EC) treatment.

**Study design:**

This study evaluated the effect and usefulness of MSM in combinatory therapy with known drug doxorubicin (DOX).

**Methods:**

The effect of combinational treatment of MSM and DOX on the induction of apoptosis was evaluated in EC cell lines (ISHIKAWA, MFE-296, MFE-280).

**Results:**

We observed that MSM itself induces apoptosis in EC cell lines, and pre-treatment with MSM for 24 h increases the sensitivity of EC cells to DOX-induced apoptosis and DNA damage and that effect might be regulated by p42/44 (Erk1/2) MAPK and Akt (protein kinase B).

**Conclusion:**

These results for the first time show that MSM might act as a sensitizer of EC cells to known drugs, for which EC cells quickly acquire resistance.

**Figure:**
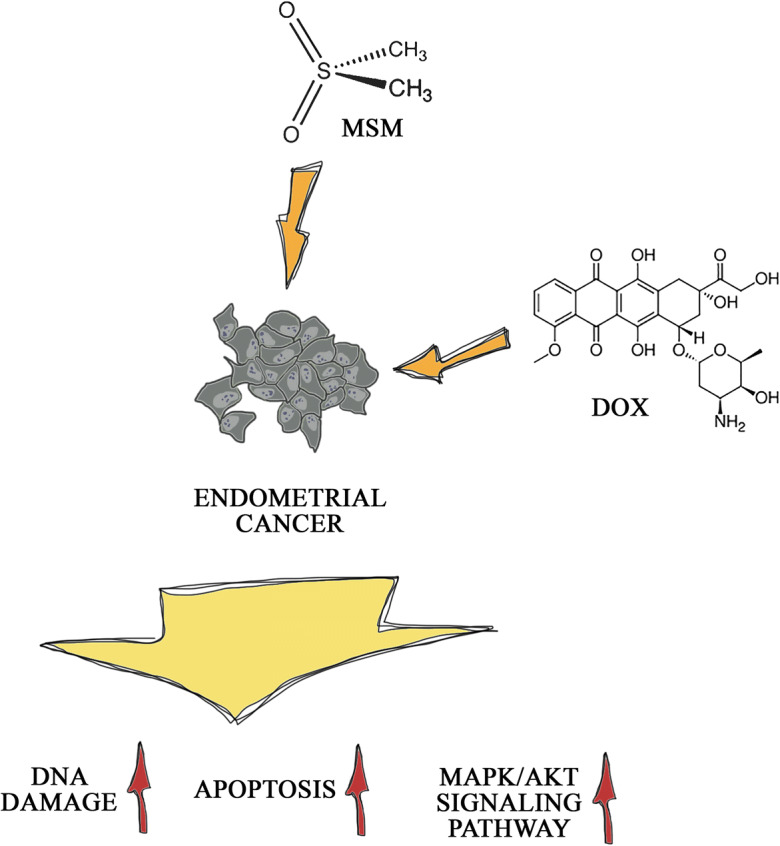
Graphical abstract

**Electronic supplementary material:**

The online version of this article (10.1007/s10565-020-09542-4) contains supplementary material, which is available to authorized users.

## Introduction

One of the most common diseases is cancer. It is estimated that approximately 40% of human population will be diagnosed with cancer in some point of their life. Cancer treatment becomes more effective; moreover, early diagnosis and effective treatments increase the number of individuals living beyond cancer diagnosis each year (Stewart et al. 2019). Endometrial cancer (EC) is the commonest gynecologic malignancy and constitutes the fourth of the commonest cancers in women in Europe (Ricceri et al. 2017). Type I of EC constitutes 80–90% of all ECs. It is associated with increased estrogen level, obesity, and young age of women and is characterized by a good prognosis of patients (Gupta 2017). The treatment of EC varies due to the grade, stage, and histology of it. Initial stages of EC are in most cases curable, and treatment involves hysterectomy, salpingo-oophorectomy, and radiotherapy. A deep myometrial invasion, cervical stromal invasion, lymphovascular invasion, tumor histology, and extrauterine progression are the main risk factors for recurrence of EC (Nomura et al. 2019a). In patients with advanced disease postoperative systemic chemotherapy, cisplatin, paclitaxel, and doxorubicin (DOX) (Morice et al. 2016) were shown to have a survival benefit as compared to radiotherapy (Nomura et al. 2019b). DOX is a cytostatic drug obtained from *Streptomyces peucetius* fungi used for the treatment of solid tumors and hematologic cancers since the 1960s (Mathias et al. 2019). DOX antitumor properties are based on intercalation to DNA and through that reduction of cells proliferation (Stewart et al. 2019). DOX is believed to be most active in the S phase, although it is a cell cycle-nonspecific drug. DOX intercalates to the DNA double helix, but its anticancer activity is the result of the induction of free radical formation and from topoisomerase II-dependent DNA cleavage (Nitiss 2009). The combinational therapy with at least two different chemotherapeutics increases response of cells up to 75%; nevertheless, the efficacy of the therapy is limited due to acquired or intrinsic cell resistance (Bae-Jump et al. 2009).

More research studies are nowadays focused on the usage of natural products with low overall toxicity in various cancers, including colorectal carcinoma, which is the third most common cancer in the world. Methylsulfonylmethane (MSM) for the last 20 years is especially known from alleviation of arthritic symptoms, supporting joints flexibility and health (Karabay et al. 2016a). MSM known as methyl sulfone or DMSO_2_ is a naturally found sulfur compound in dietary products. It occurs naturally in very low concentrations in fruits, vegetables, and milk (Cheleschi et al. 2018). Due to its low overall toxicity in humans, MSM is considered generally recognized as safe (GRAS) by the Food and Drug Administration (FDA) on July 11, 2007. MSM is mostly used as a drug supplement in treatment of inflammation in osteoarthritis (Brien et al. 2011), gastric mucosal injury (Amirshahrokhi and Khalili 2017), and even obesity-related metabolic disorders (Sousa-Lima et al. 2016).

Recently, MSM is also believed to possess anticancer effect in prostate cancer, breast (Caron et al. 2013), or bladder (Joung et al. 2014). Its apoptotic-inducing and anti-inflammatory characteristics might have a beneficial effect in many types of cancers. MSM is reported to decrease the invasiveness of cells via modulation of vascular endothelial growth factor (VEGF) in breast cancer cells (Lim et al. 2012) as well be tested in combinational therapy with Janus kinase 2 (Jak2) inhibitor in bladder cancer (Joung et al. 2014). A combinational therapy of MSM (200 mM) and tamoxifen was reported to inhibit breast cancer tumor growth and metastasis, both in vitro and in vivo by modulation of Jak2 and transducer and activator of transcription 5b (STAT5b) pathway. Deregulation of cell proliferation and apoptotic pathways is considered the main cause of tumorigenesis; accumulation of cancer cells; resistance to chemotherapeutic drugs; and angiogenesis, invasion, and metastases (Karabay et al. 2016a).

Taking into consideration the properties of MSM as well as the fact that in most cases of EC in which chemotherapeutics are needed, the combinational therapy possesses beneficial effect (Bae-Jump et al. 2009), and MSM might serve as an interesting agent. In that case, the aim of this study is to verify if MSM might induce apoptosis in EC cells and acts as a sensitizer of cells to treatment with DOX to increase its apoptotic effect.

## Materials and methods

### Cell culture and treatment

Three different EC in vitro models were used: ISHIKAWA cells derived from well-differentiated adenocarcinoma, representing histological grade 1 (G1); MFE-296 cells derived from moderately differentiated adenocarcinoma, representing histological grade 2 (G2); and MFE-280 cells derived from poorly differentiated endometrial carcinoma, representing histological grade 3 (G3). All cell lines were obtained from the European Collection of Authenticated Cell Cultures (EACC) (Sigma-Aldrich) and cultured in standard conditions (37 °C, 5% CO_2_) in minimal essential medium (MEM) (Thermo Fisher Scientific Inc./Life technologies) supplemented with 10% heat-inactivated fetal bovine serum (FBS) (Thermo Fisher Scientific Inc./Life technologies), 1 mM sodium pyruvate (Thermo Fisher Scientific Inc./Life technologies), 10 mM HEPES buffer (Thermo Fisher Scientific Inc.), and antibiotics (penicillin 50 U/mL; streptomycin 50 μg/mL; neomycin 100 μg/mL) (Thermo Fisher Scientific Inc./Life technologies). MEM was used as experimental medium without phenol red, FBS, and antibiotics. For ISHIKAWA cells, medium was supplemented with additional 1% of nonessential amino acids (Thermo Fisher Scientific Inc./Life technologies).

MSM (Sigma-Aldrich) was dissolved as stock solution (1.06 M) in experimental medium before each experiment. Stock solution of DOX (Sigma-Aldrich) (58.6 mM) was prepared in H_2_O DEPC (Thermo Fisher Scientific); working solution of 1 mM was prepared before use in experimental medium. In all experiments, cells treated with experimental medium were used as control (Cnt).

### Cell viability

The ability of cells to reduce AlamarBlue® reagent (Thermo Fisher Scientific Inc./Life technologies) was used as the viability indicator. The concentrations of MSM and DOX were based on literature (Gentilin et al. 2017), and previous experiments with MSM (Kowalska et al. 2018). Cells (20–25 × 10^3^ per well) were seeded on a 96-well plate and 1 day after seeding were treated with the experimental medium containing MSM for 24 h. Then, the medium was exchanged for DOX containing medium for additional 24 h. Cells were treated for last 24 h with MSM or DOX. Four hours before the end of incubation time, 10 μl AlamarBlue® reagent were added to each well and incubated for additional 4 h. Absorbance was measured at 570 nm and 600 nm as background in a EL808IU BioTek microplate reader (BioTek Instruments, Inc.). The results were expressed as the percentage of control (Cnt) cells. The results are presented as mean ± SE of ≥ 4 replicates.

### Cell cycle

The percentage of cells in phase subG0/G1, G0/G1, S, and G2/M of the cell cycle was determined with a Muse® Cell Cycle Assay Kit (Merck Millipore). Cells (0.8 × 10^6^) were seeded on 6-well plates and cultured in standard conditions to reach 90% confluence. Then, cells were treated with experimental media. After that time, cells were trypsinized, and a cell cycle assay was conducted according to manufacturer’s recommendations. Cells were analyzed on Muse® Cell Analyzer and standardized to Cnt cells. The results are expressed as a percentage of cells in subG0/G1, G0/G1, S, and G2/M cell cycle phase cells. The experiment was conducted in triplicate.

### Apoptosis

Apoptotic cells were stained with annexin V and 7-AAD with Muse® Annexin V Dead Cells Kit. Cells (0.8 × 10^6^) were seeded on 6-well plates and cultured in standard condition to reach 90% confluence and then treated with experimental media. Then, the experiment was conducted according to manufacturer’s recommendation. The results are expressed as a % of gated cells of three, independent experiments.

### DAPI staining

DAPI staining was conducted to evaluate the morphology of nuclei and determine possible DNA damage. Cells (20–25 × 10^3^) were seeded on 96-well plates and treated with experimental media. Then, cells were harvested with 4% paraformaldehyde (PFA, Sigma-Aldrich) in PBS for 20 min, next washed three times with PBS, incubated for 5 min with 0.1 μg/ml DAPI (Sigma- Aldrich) solution in PBS, and once again washed and photographed with Floid® Cell Imaging Station (Thermo Fisher Scientific).

### Mitochondrial potential

Muse® MitoPotential Assay (Merck Millipore) was used to evaluate the changes in mitochondrial potential of cells associated with the process of apoptosis. The staining is based on MitoPotential Dye—a cationic, lyophilic dye which detects changes in the mitochondrial membrane: a decrease in fluorescence is associated with depolarization of mitochondria. Simultaneous use of 7-AAD allows evaluation of cell membrane integrity. Cells (0.4 × 10^5^) were seeded on 12-well plates and left to reach 90% confluence. Then, the cells were treated with experimental medium. The assay was performed as recommended by the manufacturer. The probes were standardized against control probes. The experiment was repeated three times.

### DNA damage

Muse® Multicolor DNA Damage Kit was used to evaluate the DNA damage caused by MSM and DOX. The kit is based on the detection of the activation of ATM and H2A.X. Cells were seeded as previously on 12-well plates and treated with experimental medium. Then, the protocol was conducted according to producer’s recommendations. The experiment was standardized to control cells in three independent replicates.

### Real-time qPCR (RTqPCR)

cDNA was synthesized from 5 μg of total RNA using ImProm RT-IITM reverse transcriptase (Promega) according to the manufacturer’s instructions. A LightCycler 96 (Roche) was used to perform the RTqPCR reaction with 2 μl of cDNA. Primers were designed and verified using Primer-BLAST (National Institutes of Health) (Table 1). The analysis was performed using a DFS-Taq DNA Polymerase kit (BIORON). The human reference RNA (Stratagene, San Diego, CA, USA) was used as a calibrator for each reaction. The relative expressions of genes were normalized to three reference genes: ribosomal protein S17 (RPS17), ribosomal protein P0 (*RPLP0*), and histone H3.3A (*H3F3A*). The qPCR array data was analyzed using the ΔΔCt method. The results were obtained in duplicate from three repeats of the experiment and expressed as relative expression.

**Table 1:** Sequences of primers used in RTqPCR

Gene	Sequence (5′-3′)	Product size [bp]
*CDKN1A*	For GACAGATTTCTACCACTCCAA Rev. CTGAGACTAAGGCAGAAGAGT	134
*HIF-1α*	For TTACTCATCCATGTGACCATGA Rev. AGTTCTTCCTCGGCTAGTTAG	140
*SOD1*	For GCGTGGCCTAGCGAGTTAT Rev. ACACCTTCACTGGTCCATTACT	114
*PARP1*	For TCTTCAAGAGCGATGCCTATT Rev. TGAGGTAAGAGATTTCTCGGAA	129
*RPS17*	For AAGCGCGTGTGCGAGGAGATCG Rev. TCGCTTCATCAGATGCGTGACATAACCTG	87
*RPLP0*	For ACGGATTACACCTTCCCACTTGCTAAAAGGTC Rev. AGCCACAAAGGCAGATGGATCAGCCAAG	69
*H3F3A*	For AGGACTTTAAAAGATCTGCGCTTCCAGAG Rev. ACCAGATAGGCCTCACTTGCCTCCTGC	74

*CDKN1A* cyclin-dependent kinase inhibitor 1A, *HIF-1α* hypoxia inducible factor 1α, *SOD1* superoxide dismutase 1, *PARP1* poly [ADP-ribose] polymerase 1, *RPS17* ribosomal protein S17, *RPLP0* ribosomal protein P0, *H3F3A* histone H3.3A, *bp* base pair

### Western blot

Cells (2 × 10^6^) were seeded on Petri dishes and induced as described previously. After the incubation time, the cells were detached, and the protein was isolated with RIPA buffer (Sigma-Aldrich) supplemented with protease and phosphatase inhibitor cocktails (Sigma-Aldrich) and 1 mM PMSF (Sigma-Aldrich). Protein concentration was determined by DirectDetect® (Merck Millipore). Thirty micrograms of proteins were separated on 12% polyacrylamide gel (120 V) and transferred on PVDF membranes with wet transfer (100 V, 400 mA, 70 min). Membranes were then blocked in 5% fat-free milk in TBST buffer prior to overnight incubation in 4 °C in the primary antibodies, anti-Akt (#9272S), anti-phospho-Akt (#4060S), anti-p44–42 (Erk1/2, #4695S), anti-phospho-p44–42 (#4370S), anti-SOD1 (#71G8), anti-cleaved PARP1 (#5625, Cell Signaling Technology), and anti-GAPDH (1:1000, sc-59,540, SantaCruz Biotechnology), as a reference. After incubation, the membranes were washed three times with TBST buffer and incubated with secondary antibodies (1:15000, Sigma-Aldrich) for 4 hours at 4 °C. The membranes were washed once again, and the bands were visualized by using Novex® AP Chromogenic Substrate (BCIP/NBT) (Thermo Fisher Scientific). Densitometric analysis was conducted with ImageJ. The experiment was conducted in triplicate.

### Statistical analysis

The results were expressed as a mean ± SE and analyzed with one-way ANOVA. Values below *p =* 0.05 were considered statistically significant. GraphPad Prism (GraphPad Software) was used for all statistical analyses.

## Results

### MSM decreases viability of EC cells in a dose-dependent manner

**Fig. 1:**
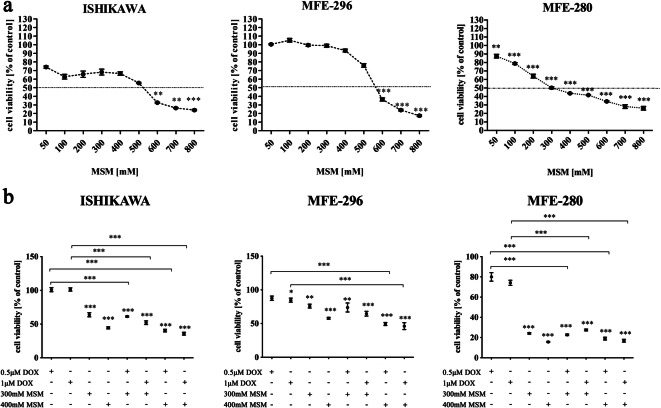
**a** MSM decreases viability of EC cells in a dose-dependent manner. The viability of cells was evaluated after exposure to MSM for 24 h with AlamarBlue® reagent. The results are expressed as a % of control cells (100% viability for non-treated cells). *p < 0.05* was considered statistically significant. **p < 0.01, ***p < 0.001. *MSM* methylsulfonylmethane. **b** Pre-treatment with MSM before DOX treatment decreases the viability of EC cells as compared to DOX treatment alone. Cells were pre-treated with MSM or experimental medium for 24 h before DOX treatment for 24 h. The viability was determined with AlamarBlue® reagent and expressed as % of Cnt cells (100% viability for non-treated cells). *p < 0.05* was considered statistically significant. *p < 0.05, **p < 0.01, ***p < 0.001 as compared to Cnt. *MSM* methylsulfonylmethane, *DOX* doxorubicin, *Cnt* control

Firstly, we evaluated the effect of MSM on EC cells and observed that MSM decreases the viability of EC cells in a dose-dependent manner (Fig. 1a). ISHIKAWA cell line, which represents the well differentiated EC, presented the highest sensitivity to MSM as compared to moderately differentiated MFE-296 and poorly differentiated MFE-280. A significant decrease in the viability of ISHIKAWA and MFE-296 cell lines was observed for doses of MSM below 600 mM (**p < 0.01, ***p < 0.001). In case of MFE-280, a statistically significant decrease in cell viability was observed for 300 mM MSM and higher doses (**p < 0.01). For the rest of experiments, the concentrations of 300 mM and 400 mM of MSM were chosen. Both doses of MSM are for most of the cell lines above the IC_50_ (half inhibitory value): for ISHIKAWA IC_50_ = 538.6 mM, MFE-296 IC_50_ = 551.1 mM MSM, and for MFE-280 IC_50_ = 380 mM MSM, based on AlamarBlue assay results (GraphPad Prism software).

Next, the viability of EC cells was once again evaluated with using MSM before DOX treatment to verify if MSM might increase the toxic effect of DOX on EC cells (Fig. 1b). Pre-treatment of ISHIKAWA and MFE-280 cells with MSM in both doses caused a statistically significant decrease in cells viability as compared to DOX treatment only (***p < 0.001). In MFE-296, pre-treatment with 400 mM MSM caused significant decrease in cell viability (***p < 0.001). Although no significant changes were observed between MSM treatment alone and pre-treatment with MSM and treatment with DOX, a decrease in the cell viability was observed.

### MSM increases DOX-induced apoptosis in EC cells

**Fig. 2:**
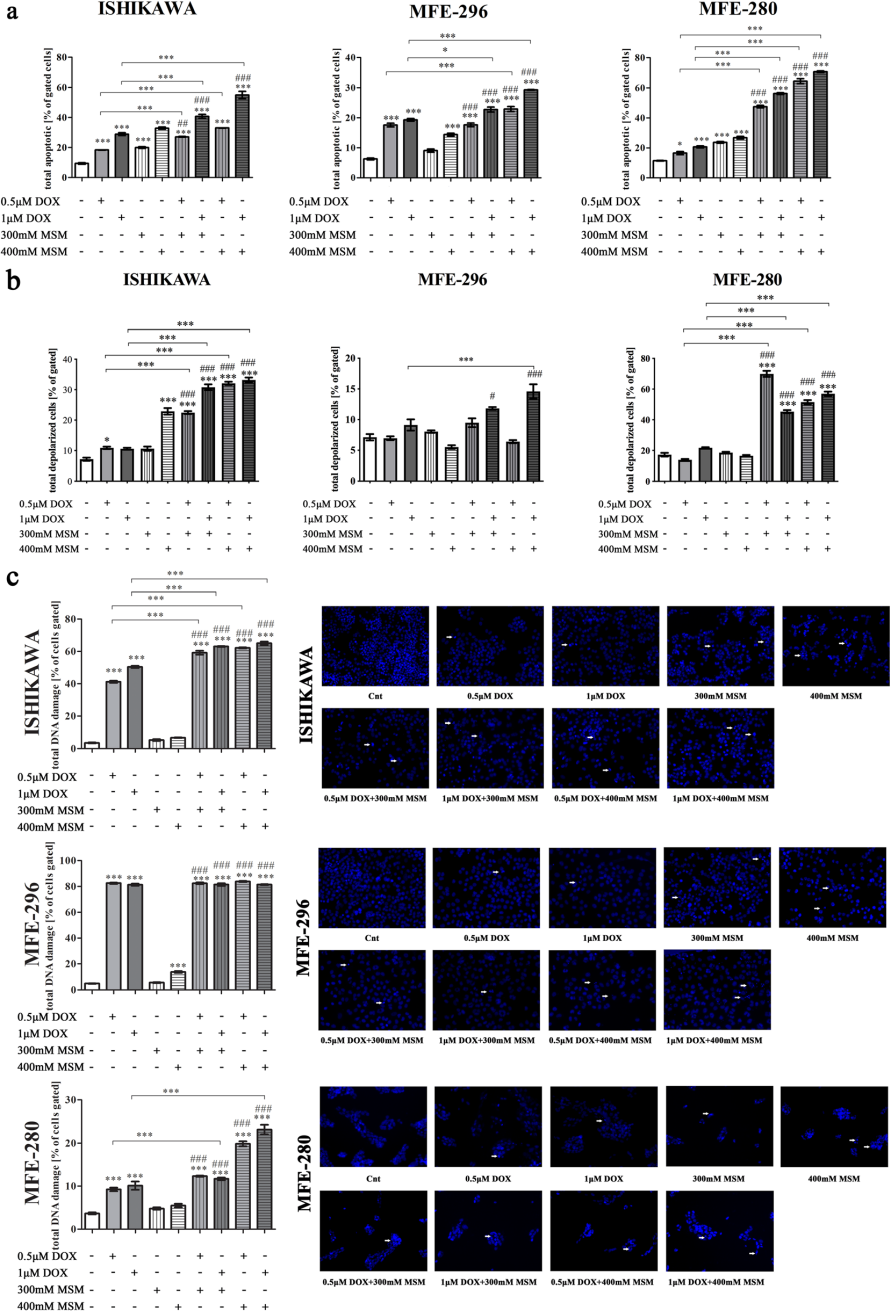
**a** MSM increases DOX-induced apoptosis in EC cells. The results are expressed as % of gated cells. *p < 0.05* was considered statistically significant. *p < 0.05, ***p < 0.001 as compared to Cnt. ##*p < 0.01*, ###*p < 0.001* as compared to MSM treatment in the same dose. *MSM* methylsulfonylmethane, *DOX* doxorubicin, *Cnt* control. **b** Pre-treatment with MSM before DOX treatment increases mitochondrial potential in EC cells. The results are expressed as % of gated cells. *p < 0.05* was considered statistically significant. *p < 0.05, ***p < 0.001 as compared to Cnt. ##*p < 0.01*, ###*p < 0.001* as compared to MSM treatment in the same dose. *MSM* methylsulfonylmethane, *DOX* doxorubicin, *Cnt* control. **c** MSM increases DOX-induced DNA damage in EC cells. DNA damage was evaluated with Muse® Multicolor DNA damage. The results are expressed as % of cells gated. *p < 0.05* was considered statistically significant. ***p < 0.001 as compared to Cnt. ###*p < 0.001* as compared to MSM treatment. In DAPI staining with white arrows are marked fragmentated nuclei of cells. *MSM* methylsulfonylmethane, *DOX* doxorubicin, *Cnt* control

Next, the induction of apoptosis was evaluated (Fig. 2a) by staining both early and late apoptotic cells. We observed that MSM induces apoptosis (***p < 0.001) in all EC cell lines. Moreover, pre-treatment of cells with MSM before DOX treatment significantly increased the apoptosis in EC cells (*p < 0.05, ***p < 0.001). The induction of apoptosis was also significantly higher than induced by MSM alone (##*p < 0.01*, ###*p < 0.001*). Interestingly, the highest increase in MSM + DOX-induced apoptosis as compared to MSM and DOX treatment alone was observed for MFE-280 cell lines, which represents the poorly differentiated EC I stage in vitro model. Next, we evaluated the changes in mitochondrial transmembrane potential which might be associated with observed induction of apoptosis (Fig. 2b.). The induction of apoptosis in EC cells was associated with increased mitochondrial potential in ISHIKAWA and MFE-280 cells for all tested combinatory doses of DOX and MSM as compared to DOX (***p < 0.001) and MSM treatment alone (###*p < 0.001*). A statistically significant increase in the mitochondrial potential in MFE-296 cells was observed for the highest tested combinatory dose of MSM and DOX (***p < 0.001).

The induction of apoptosis was also associated with DNA damage (Fig. 2c). It was observed that DOX itself has a high effect on DNA damage in all EC cell lines (***p > 0.001). Pre-treatment with MSM before DOX exposure significantly increased DNA damage in ISHIKAWA (***p < 0.001) and MFE-280 for higher dose of MSM (***p < 0.001). No significant increase in DNA damage was observed after MSM pre-treatment in MFE-296 cells, probably due to high increase in DNA damage caused by DOX itself. In all tested cell lines, pre-treatment with MSM before DOX treatment increased the number of cells with damaged DNA as compared to MSM treatment alone (###*p < 0.001*). DNA fragmentation was visualized with DAPI staining (Fig. 2c). In all cell lines, pre-treatment with MSM before DOX treatment caused visible increase in the number of fragmented nuclei marked with white arrows.

**Fig. 3:**
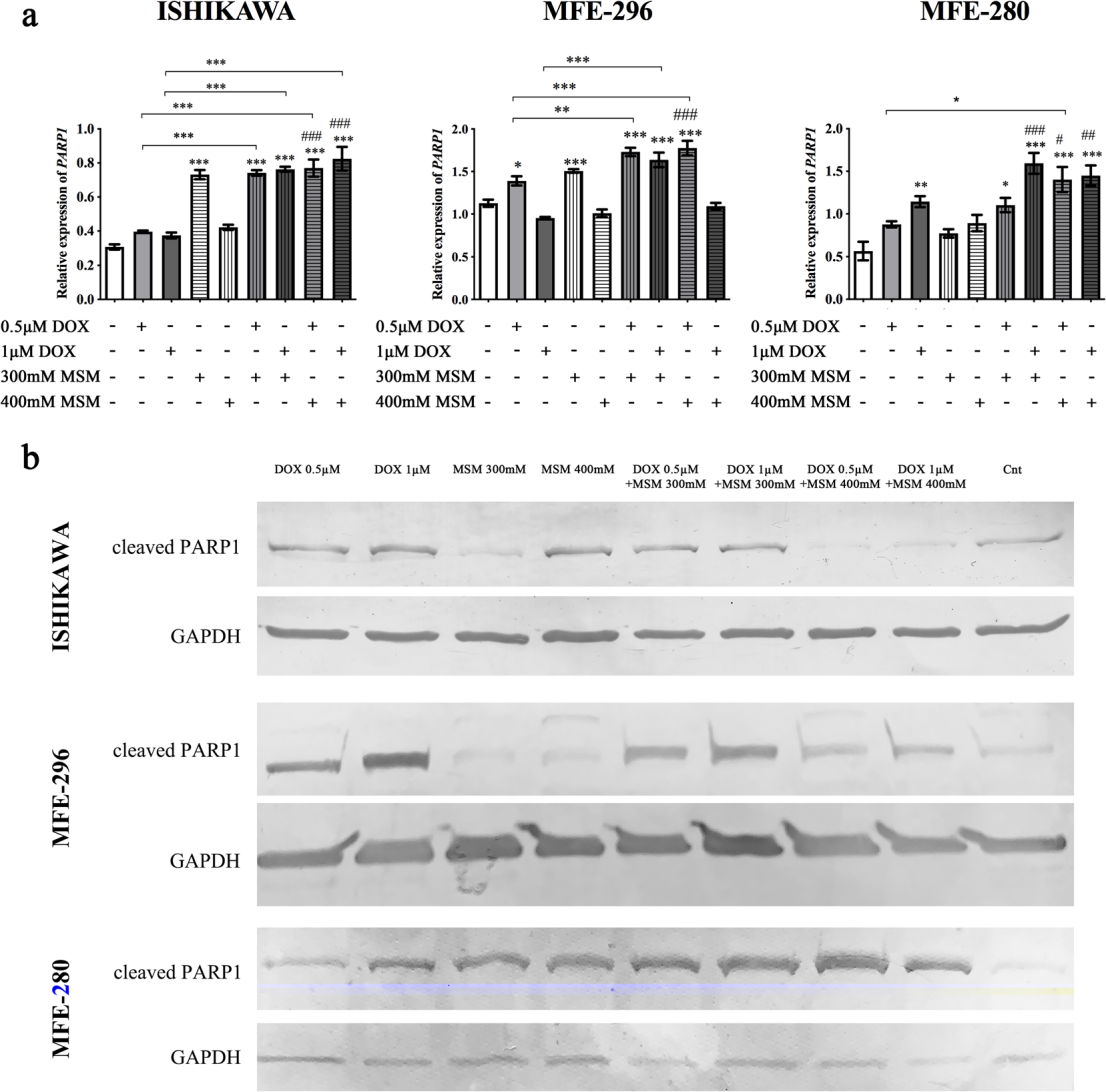
**a** The expression of *PARP1* after MSM pre-treatment before exposure to DOX. The expression was evaluated with RTqPCR. The results are expressed as a relative expression. *p < 0.05* was considered statistically significant. *p < 0.05, **p < 0.01, ***p < 0.001 as compared to Cnt. #*p < 0.05*, ###*p < 0.001* as compared to MSM-treated cells. **b** The representative results of Western blot evaluation of cleaved PARP1 form. *MSM* methylsulfonylmethane, *DOX* doxorubicin, *Cnt* control

A statistically significant increase in the expression of *PARP1* (Fig. 3a) was observed simultaneously with the induction of apoptosis. In all cell lines with the exception of MFE-296 for the highest dose of MSM and DOX, the expression of *PARP1* was increased after pre-treatment with MSM and treatment with DOX, as compared to control (***p < 0.001). For ISHIKWA cells, the highest dose of MSM in pre-treatment caused a statistically significant increase in the expression of *PARP1* as compared to DOX as well as MSM treatment alone (***p < 0.001, ###*p < 0.001*, respectively). Similar effect was observed for MFE-280 and MFE-296 for both doses of MSM. We also evaluated the expression of cleaved PARP1 on protein level (Fig. 3b). The 89-kDa product of cleavage of PARP-1 is reported to be a cause of caspase-3 and caspase-7 activity in cells (Chaitanya et al. 2010). We observed that only in case of MFE-280, an increase in the expression of cleaved PARP1 was higher after treatment with MSM and DOX as compared to DOX and MSM treatment alone (Table 2). An increase in the expression of cleaved PARP1 after pre-treatment with MSM and then treatment with DOX was also observed in MFE-296, however was lower than observed for DOX treatment alone. Interestingly, in the case of ISHIKAWA cells, the highest dose of MSM in pre-treatment caused a decrease in the expression of cleaved PARP1.

**Table 2:** The relative expression of cleaved PARP1 (89 kDa) obtained in Western blot analysis

Treatment									
Relative expression of cleaved PARP1	0.5 μM DOX	1 μM DOX	300 mM MSM	400 mM MSM	0.5 μM DOX + 300 mM MSM	1 μM DOX + 300 mM MSM	0.5 μM DOX + 400 mM MSM	1 μM DOX + 400 mM MSM	Cnt
	ISHIKAWA								
	0.998	1.549	0.738	1.385	1.028	1.336	0.879	0.841	1.000
	MFE-296								
	3.503	5.650	1.073	0.928	2.694	3.149	1.823	2.671	1.000
	MFE-280								
	1.624	2.632	2.077	2.203	3.571	3.123	5.268	6.624	1.000

The results are expressed as mean of fold change of the expression normalized to control sample. The experiment was conducted in duplicate

*MSM* methylsulfonylmethane, *DOX* doxorubicin, *Cnt* control, *PARP1* poly (ADP-ribose) polymerase-1

**Fig. 4:**
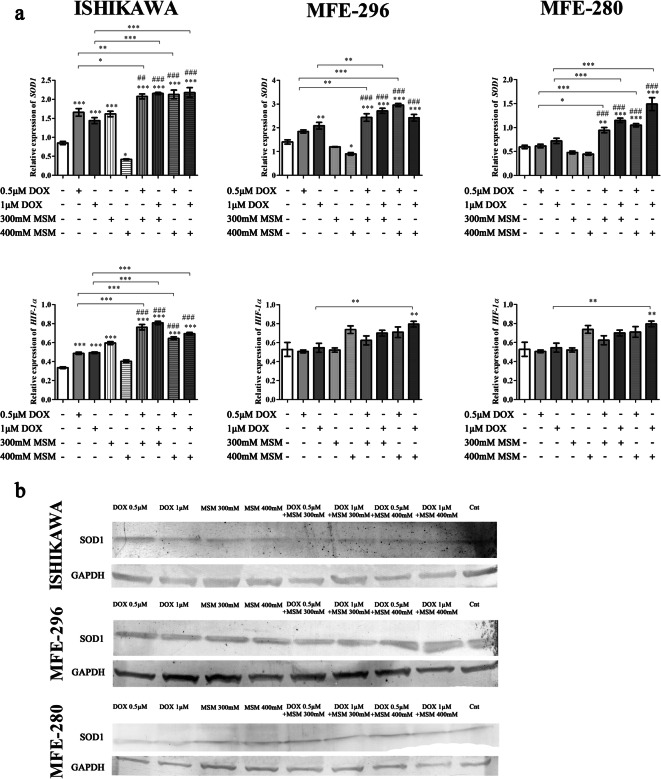
**a** The expression of *SOD1* and *HIF-1α* after MSM and DOX treatment. The expression was evaluated with RTqPCR. The results are expressed as a relative expression. **b** The expression of SOD1 on protein level. The expression was evaluated with Western blot and presented as representative results. *p < 0.05* was considered statistically significant. *p < 0.05, **p < 0.01, ***p < 0.001 as compared to Cnt. ##*p < 0.01*, ###*p < 0.001* as compared to MSM-treated cells. *MSM* methylsulfonylmethane, *DOX* doxorubicin, *Cnt* control

DNA damage and induction of apoptosis might be associated with oxidative stress in cells; based on this assumption, the next stage of the study evaluated the expression of *SOD1* and *HIF1α*. We observed that MSM and DOX modulate the expression of *SOD1* and *HIF1α* expression. The expression of *SOD1* was decreased in all EC cell lines after treatment with higher dose of MSM and increased after treatment with DOX (Fig. 4a). In all EC cells, pre-treatment with MSM before DOX exposure caused a significant increase in the expression of *SOD1* as compared to Cnt (***p < 0.001) as well as MSM (###*p < 0.001*) and DOX (*p < 0.05, **p < 0.01, ***p < 0.001) treatments alone. The expression of SOD1 was also evaluated on the protein level (Fig. 4b), and similar tendency was observed. Comparable effect was observed for the expression of *HIF-1α* in ISHIKAWA cells and highest dose of MSM in MFE-296 cells. In MFE-280, the changes in the expression of *HIF-1α* were not significant with the exception of pre-treatment of cell with 400 mM MSM before treatment with 1 μM of DOX (**p < 0.01).

**Fig. 5:**
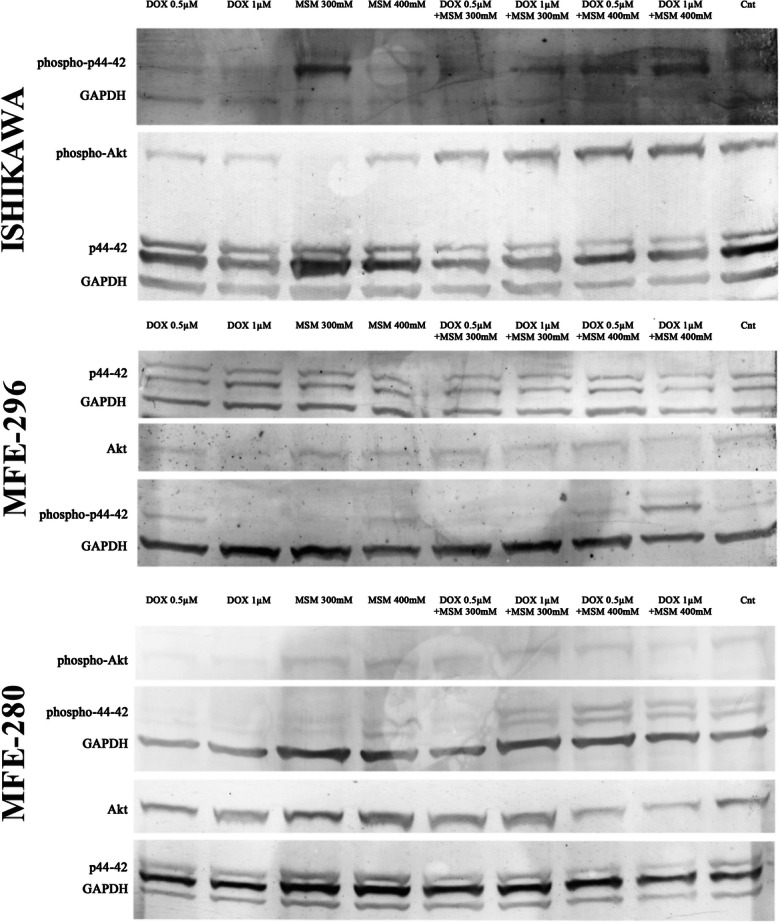
The expression of Akt and p44–42 after DOX and MSM exposure in EC cell lines. The expression was evaluated with Western blot and presented as representative results. *MSM* methylsulfonylmethane, *DOX* doxorubicin, *Cnt* control

MAPK signaling plays a crucial role in the sensitivity of cells to anticancer therapies via regulation of growth, differentiation, and apoptosis (Sui et al. 2014). Moreover, due to the known modulation of MAPK and Akt signaling pathways by MSM, we also evaluated the expression of Akt, p-Akt, p44–42, and p-p44–42 on the protein level. In all EC cell lines, we observed an increase in the expression of p-p44–42 after pre-treatment with MSM before DOX exposure as compared to DOX as well as MSM treatment alone (Fig. 5). The expression of p-Akt was also increased in ISHIKAWA and MFE-280 cell lines, whereas in MFE-296 was slightly detectable; nevertheless, the expression of Akt was decreased.

**Fig. 6:**
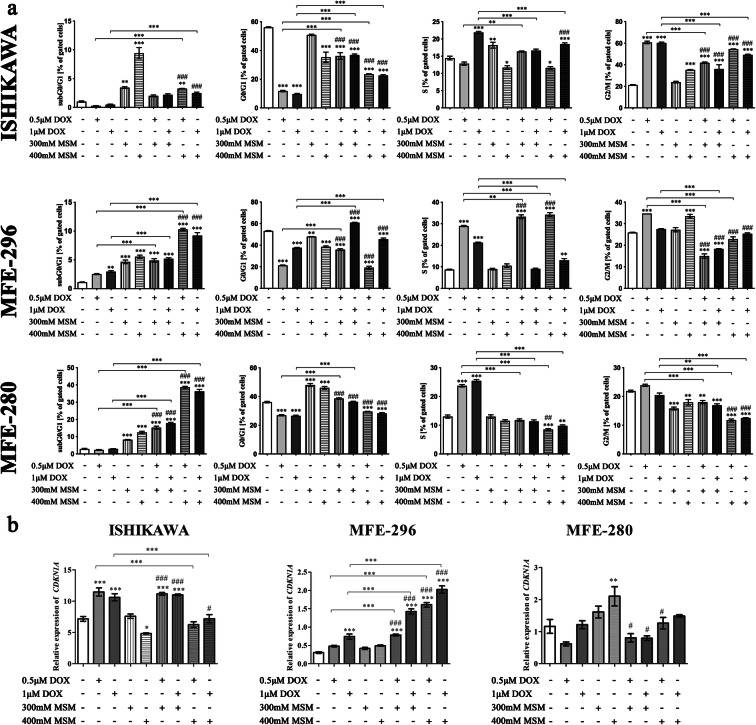
**a** Cell cycle distribution after pretreated with MSM EC cells before DOX exposure. The cell cycle distribution is expressed as % of gated cells. *p < 0.05* was considered statistically significant. *p < 0.05, **p < 0.01, ***p < 0.001 as compared to Cnt. #*p < 0.05*, ##*p < 0.01*, ###*p < 0.001* as compared to MSM treatment. *MSM* methylsulfonylmethane, *DOX* doxorubicin, *Cnt* control. **b** Expression of *CDKN1A* after DOX, MSM, and MSM + DOX treatment in EC cells. The expression of *CDKN1A* was evaluated in RTqPCR. The results are expressed as a relative expression. *p < 0.05* was considered statistically significant. *p < 0.05, **p < 0.01, ***p < 0.001 as compared to Cnt. #*p < 0.05*, ###*p < 0.001* as compared to MSM treatment. *MSM* methylsulfonylmethane, *DOX* doxorubicin, *Cnt* control

In the next part of experiments, the distribution of cell cycle was evaluated (Fig. 6a). In ISHIKAWA cells, we observed the highest increase in G2/M cell cycle phase after exposure of cells to DOX. MSM also caused increase in the number of cells in G2/M cell cycle phase in the highest concentration as compared to control cells, and pre-treatment with MSM before DOX exposition caused lower increase as compared to DOX alone (***p < 0.001). In the case of MFE-296 cells, the cell cycle seems to be stopped at S phase after exposure to two doses of MSM before 0.5 μM DOX treatment, and this effect was higher as observed for DOX and MSM treatment alone (***p < 0.001 and ###*p < 0.001*). Interestingly, in MFE-280, DOX itself caused increase in the number of cell in S cell cycle phase (***p < 0.001), whereas MSM as well as pre-treatment with MSM before DOX treatment caused significant increase in the number of cells in G0/G1 cell cycle phase (***p < 0.001), although it was decreased as compared to MSM treatment alone (###*p < 0.001*). Due to observed induction of apoptosis, the number of cells in subG0/G1 cell cycle phase was also evaluated. In ISHIKAWA cells, we observed that a significant increase in the number of cells in subG0/G1 cell cycle phase was observed only for MSM treatment alone as compared to control (**p < 0.01, ***p < 0.001, respectively). The number of cells increased significantly also for 400 mM MSM + DOX treatment as compared to DOX treatment alone (***p < 0.001, *p < 0.05, respectively, for 0.5 and 1 μM of DOX). In case of MFE-296, we observed a statistically significant increase in the number of cells in subG0/G1 cell cycle phase for MSM + DOX treatment as compared to control, DOX treatment alone, and 400 mM MSM. Similar effect was observed for MFE-280, statistically significant as compared with control, DOX and MSM treatments alone. The results indicate that pre-treatment with MSM before exposure of EC cells to DOX triggers a different cell cycle regulation, dependently on the cell line. For ISHIKAWA cells, we rather observed cell cycle arrest in S and G2/M cell cycle phases, whereas for MFE-280, the results presented here indicate cell cycle arrest in G0/G1. In case of MFE-296, it seems that the cell cycle arrest is dependent on the dose: for the lower dose of DOX and MSM-pre-treatment, we observed similar effect to ISHIKAWA cells, whereas for higher dose of MSM, the effect seems to be more similar for MFE-280.

p21, encoded by *CDKN1A* gene, is a pivotal cell cycle regulator which is often deregulated in human cancer. Its expression is increased in response to different stimuli to arrest the cell cycle and ensure genomic stability (Kreis et al. 2019).Following this observation of cell cycle modulation, the next stage evaluated the expression of *CDKN1A* (Fig. 6b). A statistically significant increase in the expression of *CDKN1A* after pre-treatment with MSM before DOX exposition was observed, as compared to DOX treatment alone (***p < 0.001) as well as MSM treatment alone (###*p < 0.001*). Different expression pattern was observed for MFE-280 cell line, for which the highest increase in the expression was presented for the highest dose of MSM (**p < 0.01) and decreasing for MSM-pre-treatment as compared to MSM treatment (#*p < 0.05*). For ISHIKAWA cells, DOX treatment significantly increased expression of *CDKN1A* as compared to Cnt (***p < 0.001), whereas the highest dose of MSM caused a significant decrease in *CDKN1A* expression. Pre-treatment with 300 mM MSM and then treatment with DOX caused similar to DOX-induced increase in the expression of *CDKN1A*, whereas higher dose caused significant decrease in the expression as compared to DOX treatment alone (***p < 0.001).

## Discussion

Both the increasing incidence and mortality rates of EC in developed countries are mostly associated with obesity and diabetes, which constitutes a barrier for surgery and chemotherapy. Taking into consideration that first-line therapies are limited for EC, the natural molecules which might serve as independent medicine or be involved in known therapies seem to be potentially useful in EC treatment (Lee et al. 2017). There is a high need of identifying new biomarkers to be a basis of personalized therapy, as well as to evaluate the potential targeted therapeutics, hormonal therapy, and immunotherapy in EC (Charo and Plaxe 2019).

As suggested before, the combinational therapy has a greater effect in comparison to one chemotherapeutic alone (Gantzer and Ray-Coquard 2018). Our study also showed that combinational therapy of MSM and DOX has a greater cytotoxic effect than DOX treatment alone. MSM was previously observed to induce apoptosis in different cell lines (Kowalska et al. 2018; Lim et al. 2012). Similarly, we observed that in EC cell lines, MSM also induced decrease in cell viability in a dose-dependent manner. Interestingly, we observed that different EC cell lines responds differently to MSM and ISHIKAWA cell line, which represents well-differentiated, estrogen responding and seems to be more sensitive to MSM-induced cell death. Similarly in our previous study, we also observed that hormone-dependent LNCaP cell lines are more sensitive than the others (Kowalska et al. 2018), indicating that hormonal sensitivity of cells might play a role in MSM-induced toxicity in cancer cells. The different response of EC cell lines might be also associated with different migratory and proliferation profile of these cell lines, as reported before (Parkes et al. 2018).

MSM was previously reported to decrease the viability of cells via modulation of cell cycle progression and induction of apoptosis (Karabay et al. 2016a). Similar results were observed by us in this study. MSM-induced apoptosis in EC cells and pre-treatment of cells with MSM significantly increased DOX-induced apoptosis in EC cells. Previously, MSM-induced apoptosis was associated with inhibiting and DNA binding of transcription factors: p53 or STAT3 and STAT5b (Lim et al. 2012). In our study, the apoptotic effect of MSM was associated with fragmentation of DNA and its damage. It was also found that the expression of *CDKN1A* was modulated. These results are similar to previous one suggesting that p53 might be a target of MSM action in cancer cells, which directly regulates the expression of *CDKN1A*, although MSM effect might be independent of the p53 presence (Karabay et al. 2016b)*.* Observed a different effect of pre-treatment of cells with MSM before DOX exposure in *CDKN1A* gene expression is also visible in different % of cell in cell cycle phases. In MFE-280 cells, the apoptotic effect of MSM and pre-treatment with MSM was clearly associated with cell cycle arrest in G0/G1 cell cycle phase, similar to the previously observed effect of MSM (Nipin et al. 2017) in gingival cancer cells, with the increase in p21 expression. Our results indicate that pre-treatment with MSM increases cell cycle arrest in G1 cell cycle phase instead of S or G2/M DOX-induced cell cycle arrest in EC cells. Similar effect was observed by Bajbouj et al. in breast and ovarian cancer cells treated with estrogen (E2) and DOX (Bajbouj et al. 2019). The increase in the number of cells in subG0/G1 cell cycle phase might confirm that the regulation of cell cycle was different for different cell lines used in this experiment. PARP1 is a nuclear enzyme which participates in DNA repair and gene transcription and acts as a key regulator of homologous recombination, which role in maintaining genomic integrity and response to chemotherapy is known (Lai et al. 2019). The increased expression of *PARP1* observed in this study indicates that pre-treatment of EC cells with MSM before DOX exposure increases DNA damage and cancer cells death. However, observed differences in the expression of cleaved PARP1 suggested that in the poorly differentiated cells, the induction of DNA damage was the highest in pre-treatment with MSM. It might also indicate that in other cell lines, the mechanism of apoptosis might differ from activation of caspase-3 and caspase-7 responsible for cutting PARP1 to 89 kDa form (Chaitanya et al. 2010). These results, different for EC cell lines, are in line with the different regulation of cell cycle progression mentioned above.

DOX was previously reported to induce DNA damage and might activate free radical to interact with molecular oxygen to generate superoxide radicals and in turn oxidative stress in cells (Ravi and Das 2004). We observed that pre-treatment of EC cells with MSM before DOX exposure increases expression of *HIF-1α* and *SOD1*. HIF-1α expression was previously reported to be increased after exposure to DOX (Mendivil-Perez et al. 2015). Moreover, Roncuzzi et al. suggested that activation of HIF-1α is involved in doxorubicin resistance in human osteosarcoma cells (Roncuzzi et al. 2014). Our results showed that pre-treatment with MSM sensitizes EC cell to DOX-induced DNA damage and oxidative stress.

In previous studies, MSM was reported to modulate the MAPK signaling pathway which plays an essential role in regulation of cell viability and apoptosis (Karabay et al. 2016a). MAPK signaling pathway and PI3K/Akt/mTOR were also reported to be modulated by DOX (Sahu et al. 2019). In that case, the expression of p44–42 and Akt was evaluated by us. We observed, similar to previous results, that MSM modulates expression of Akt and p44–42. The pre-treatment with MSM before DOX exposure caused decrease in phosphorylation of Akt in MFE-280 and increase in the expression of p-Akt in ISHIKAWA cells. The crosstalk between PI3K/Akt and MAPK signaling pathway in cancer was reported before (Cao et al. 2019). Interestingly we observed that when the phosphorylation of Akt increases, the phosphorylation of p44–42 decreases, indicating that MSM might both modulate PI3K/Akt as well MAPK signaling pathways, but this statement needs further studies to be confirmed. Interestingly, modulation of PI3K and ERK signaling pathways were shown previously to decrease the DOX-induced resistance in human carcinoma xenograft model (Satonaka et al. 2017). Based on the results of this study, MSM might serve as a promising candidate in EC treatment; however, further studies are needed to confirm this.

## Conclusion

This study for the first time showed that MSM might itself cause decrease in the viability and induce apoptosis in EC cells via modulation of cell cycle and DNA damage. Moreover, MSM might serve as a sensitizer to known and used chemotherapeutics in EC therapy. This in vitro study showed that pre-treatment of EC cells with MSM might increase DOX-induced oxidative stress and apoptosis, and this apoptotic effect might be the cause of different molecular mechanisms, dependent on the differentiation of EC cells, but further studies are needed to confirm these observations.

## Electronic supplementary material

ESM 1 (PNG 1132 kb)

High Resolution Image (TIF 4902 kb)

ESM 2 (PNG 1241 kb)

High Resolution Image (TIF 30156 kb)

## Abbreviations

7-AAD: 7-Aminoactinomycin D
Akt: Protein kinase B
ATM: Ataxia-telangiectasia mutated
CDKN1A: Cyclin-dependent kinase inhibitor 1A
DAPI: 4′,6-Diamidino-2-phenylindole
DNA: Deoxyribonucleic acid
DOX: Doxorubicin
EACC: European Collection of Authenticated Cell Cultures
EC: Endometrial cancer
FBS: Fetal bovine serum
FDA: Food and Drug Administration
GRAS: Generally recognized as safe
H2A.X: H2A histone family member X
H3F3A: Histone H3.3A
HIF-1α: Hypoxia inducible factor 1α
Jak2: Janus kinase 2
MAPK: Mitogen-activated protein kinases
MEM: Minimal essential medium
MSM: Methylsulfonylmethane
mTOR: Mammalian target of rapamycin
PARP1: Poly [ADP-ribose] polymerase 1
PBS: Phosphate-buffered saline
PFA: Paraformaldehyde
PI3K: Phosphatidylinositol-3-kinase
PMSF: Phenylmethylsulfonyl fluoride
PVDF: Polyvinylidene fluoride
RIPA: Buffer-Radioimmunoprecipitation assay buffer
RNA: Ribonucleic acid
RPLP0: Ribosomal protein P0
RPS17: Ribosomal protein S17
RTqPCR: Real-time quantitative polymerase chain reaction
SOD1: Superoxide dismutase 1
STAT5b: Transducer and activator of transcription 5b
TBST: Buffer-tris-buffered saline with Tween 20
VEGF: Vascular endothelial growth factor
