# Supplementary figures and images for: Methylsulfonylmethane sensitizes endometrial cancer cells to doxorubicin
Source: Cell Biol Toxicol. 2020 Jun 20;37(2):261–75. doi: 10.1007/s10565-020-09542-4 (PMC8012311; doi:10.1007/s10565-020-09542-4)

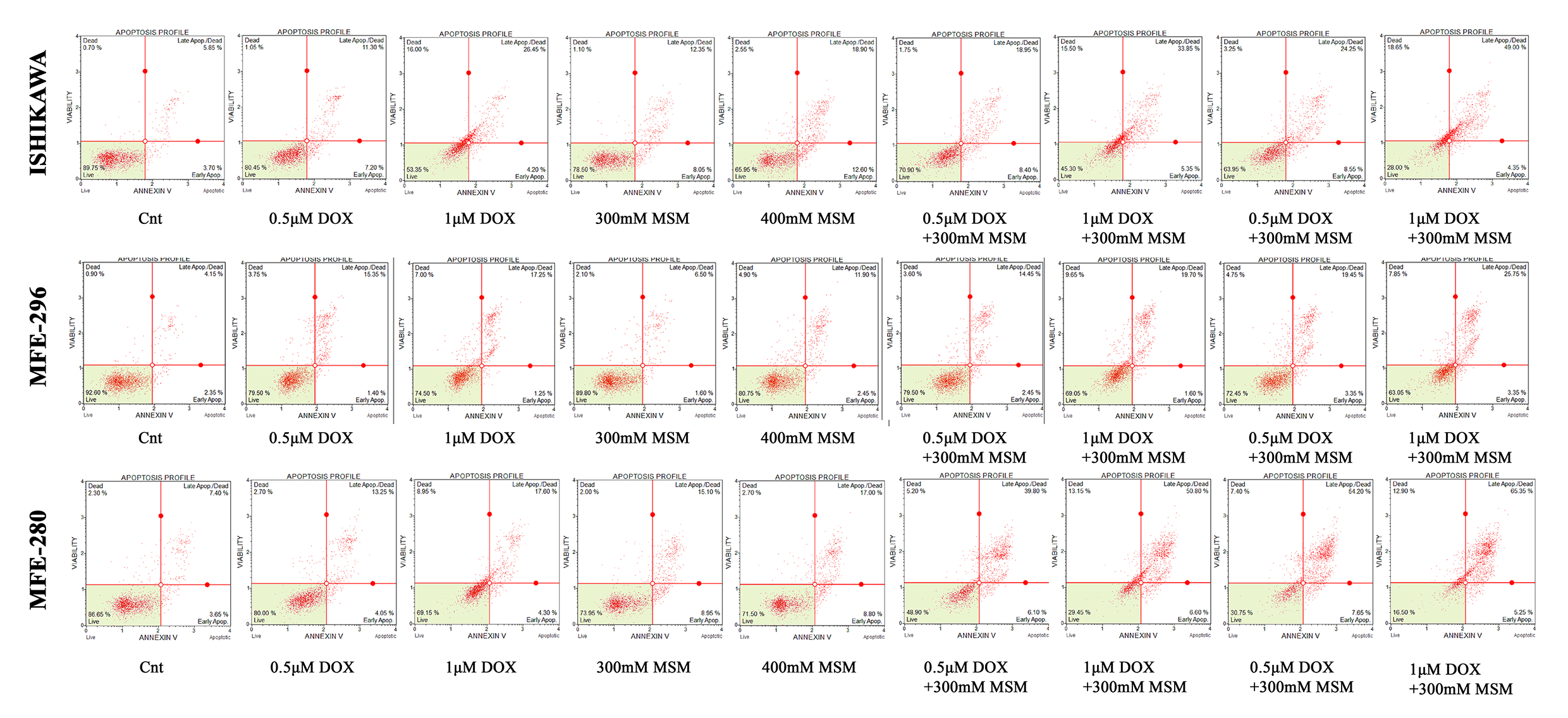

Supplement: Supplementary file 1 — (PNG 1132 kb) [file 10565_2020_9542_Fig7_ESM.png]

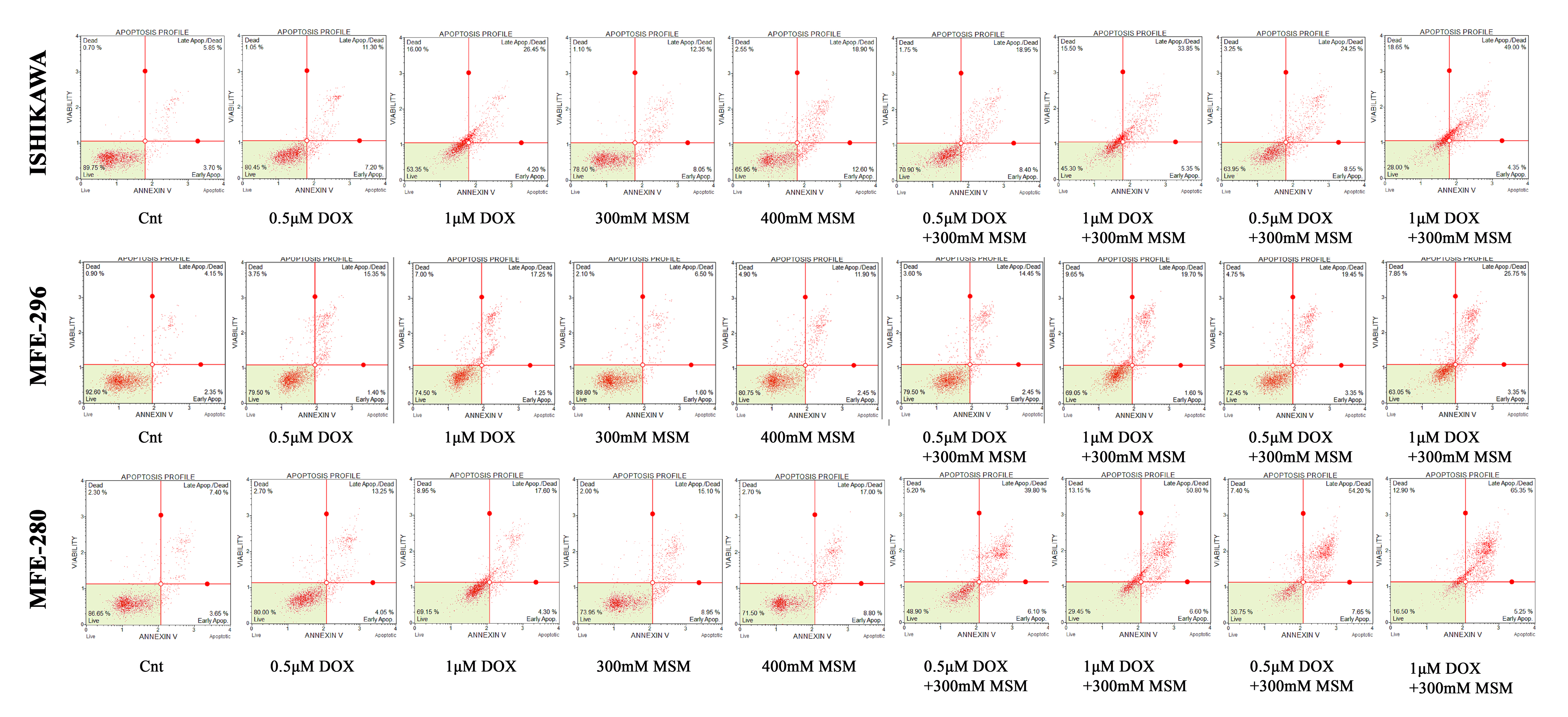

Supplement: Supplementary file 2 — High Resolution Image (TIF 4902 kb) [file 10565_2020_9542_MOESM1_ESM.tif]

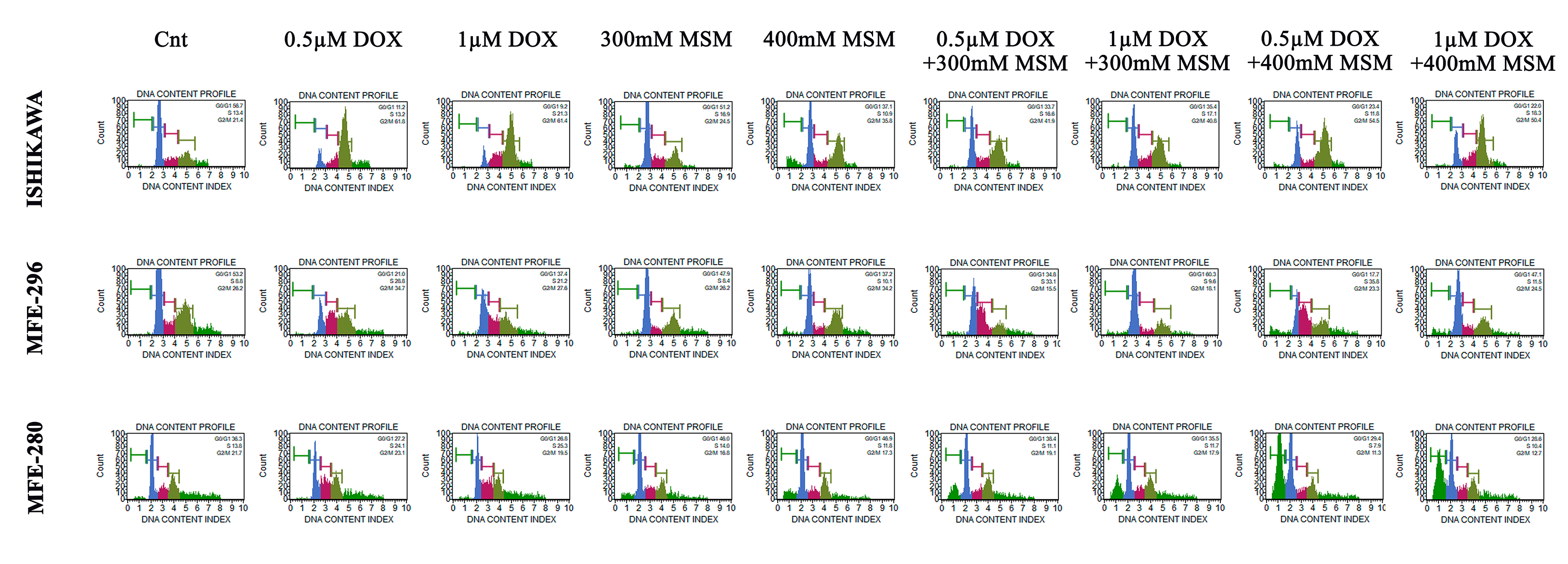

Supplement: Supplementary file 3 — (PNG 1241 kb) [file 10565_2020_9542_Fig8_ESM.png]
